# KL1 Domain of Longevity Factor Klotho Mimics the Metabolome of Cognitive Stimulation and Enhances Cognition in Young and Aging Mice

**DOI:** 10.1523/JNEUROSCI.2458-21.2022

**Published:** 2022-05-11

**Authors:** Shweta Gupta, Arturo J. Moreno, Dan Wang, Julio Leon, Chen Chen, Oliver Hahn, Yan Poon, Kenneth Greenberg, Nathaniel David, Tony Wyss-Coray, Daniel Raftery, Daniel E.L. Promislow, Dena B. Dubal

**Affiliations:** ^1^Department of Neurology and Weill Institute for Neurosciences, University of California, San Francisco, San Francisco, California 94143-1207; ^2^Department of Neurology and Neurological Sciences, Stanford University School of Medicine, Stanford, California 94305-5101; ^3^Unity Biotechnology, Inc, South San Francisco 94080; ^4^Veterans Administration Palo Alto Healthcare System, Palo Alto, California 94304-1207; ^5^Paul F. Glenn Center for the Biology of Aging, Stanford University School of Medicine, Stanford, California 94305-5235; ^6^Wu Tsai Neurosciences Institute, Stanford University School of Medicine, Stanford, California 94305-5235; ^7^Department of Anesthesiology and Pain Medicine, Mitochondria and Metabolism Center, University of Washington, Seattle, Washington 98109-4714; ^8^Fred Hutchinson Cancer Research Center, Seattle, Washington 98109-1024; ^9^Department of Lab Medicine and Pathology, University of Washington School of Medicine, Seattle, Washington 98195-7470; ^10^Department of Biology, University of Washington, Seattle, Washington 98195-1800

**Keywords:** aging, bioinformatics, cognition, klotho, metabolism, synaptic plasticity

## Abstract

Cognitive deficits are a major biomedical challenge—and engagement of the brain in stimulating tasks improves cognition in aged individuals (Wilson et al., 2002; Gates et al., 2011) and rodents (Aidil-Carvalho et al., 2017), through unknown mechanisms. Whether cognitive stimulation alters specific metabolic pathways in the brain is unknown. Understanding which metabolic processes are involved in cognitive stimulation is important because it could lead to pharmacologic intervention that promotes biological effects of a beneficial behavior, toward the goal of effective medical treatments for cognitive deficits. Here we show using male mice that cognitive stimulation induced metabolic remodeling of the mouse hippocampus, and that pharmacologic treatment with the longevity hormone α-klotho (KL), mediated by its KL1 domain, partially mimicked this alteration. The shared, metabolic signature shared between cognitive stimulation and treatment with KL or KL1 closely correlated with individual mouse cognitive performance, indicating a link between metabolite levels and learning and memory. Importantly, the treatment of mice with KL1, an endogenous circulating factor that more closely mimicked cognitive stimulation than KL, acutely increased synaptic plasticity, a substrate of cognition. KL1 also improved cognition, itself, in young mice and countered deficits in old mice. Our data show that treatments or interventions mimicking the hippocampal metabolome of cognitive stimulation can enhance brain functions. Further, we identify the specific domain by which klotho promotes brain functions, through KL1, a metabolic mimic of cognitive stimulation.

**SIGNIFICANCE STATEMENT** Cognitive deficits are a major biomedical challenge without truly effective pharmacologic treatments. Engaging the brain through cognitive tasks benefits cognition. Mimicking the effects of such beneficial behaviors through pharmacological treatment represents a highly valuable medical approach to treating cognitive deficits. We demonstrate that brain engagement through cognitive stimulation induces metabolic remodeling of the hippocampus that was acutely recapitulated by the longevity factor klotho, mediated by its KL1 domain. Treatment with KL1, a close mimic of cognitive stimulation, enhanced cognition and countered cognitive aging. Our findings shed light on how cognition metabolically alters the brain and provide a plausible therapeutic intervention for mimicking these alterations that, in turn, improves cognition in the young and aging brain.

## Introduction

Engaging the brain through cognitive stimulation is a beneficial behavior. In randomized clinical trials and observational studies, it improves cognition in individuals with mild to moderate dementia (Gates et al., 2011) and associates with reduced risk of Alzheimer's disease (AD; Wilson et al., 2002). Further, in rodents, cognitive stimulation improves synaptic plasticity, a substrate of cognition (Aidil-Carvalho et al., 2017), and improves cognition itself in models of AD (Billings et al., 2007; Martinez-Coria et al., 2015). Functional imaging studies reveal that neural and cognitive activities require energy in the form of glucose (Lundgaard et al., 2015; Magistretti and Allaman, 2015). However, whether cognitive stimulation itself alters brain metabolism remains to be defined. Since cognitive deficits are a major biomedical challenge, and modifying behavior is difficult, understanding how cognitive stimulation metabolically alters the brain could open new avenues for pharmacologic treatments that mimic beneficial behaviors.

Like cognitive stimulation, the longevity factor α-klotho (KL; Kuro-o et al., 1997) improves cognition in model organisms (Dubal et al., 2014, 2015; Leon et al., 2017; Zeng et al., 2019). KL circulates as a hormone following cleavage from its transmembrane form; it impacts glucose metabolism (Unger, 2006; Wolf et al., 2008; Château et al., 2010), FGF signaling (Urakawa et al., 2006; Lee et al., 2018), and Wnt functions (Liu et al., 2007; Zhou et al., 2013; Tang et al., 2016). Human relevance for KL in brain health is supported by findings that individuals with elevated KL, because of genetic *KLOTHO* variation, show better cognitive functions in aging and AD (Dubal et al., 2014; Yokoyama et al., 2015; Franzmeier et al., 2020; Driscoll et al., 2021). Peripheral administration of the cleaved, hormonal form of KL rapidly improves cognitive functions in mice, suggesting that it could engage metabolic pathways in the brain. KL contains two major subdomains, KL1 and KL2. KL1 is cleaved from KL and circulates as an endogenous protein (Saar-Kovrov et al., 2020). KL1 is sufficient to capitulate KL functions against tumorigenesis (Abramovitz et al., 2011; Ligumsky et al., 2015; Arbel Rubinstein et al., 2019) and cardiac stress (Wright et al., 2017). Whether KL requires a specific domain such as KL1 for cognitive enhancement and whether it could alter brain metabolism given its rapid action remain unknown.

While cognitive stimulation and KL administration—behavioral and pharmacologic interventions—both improve cognition, whether either could alter brain metabolism remains a gap in our fundamental knowledge of their potentially overlapping mechanisms. Here we show that brain engagement through cognitive stimulation induces metabolic alterations in the hippocampus that are acutely recapitulated by the longevity factor KL, mediated by its KL1 domain. Treatment with KL1, a close pharmacologic mimic of cognitive stimulation, enhanced cognition and countered cognitive aging.

## Materials and Methods

### Mice.

Mice were kept on a 12 h light/dark cycle with *ad libitum* access to food and water. Before the collection of hippocampi for metabolomics, food was removed for 4 h. Mice were anesthetized with 0.4 mg/g, i.p., avertin and perfused with room temperature (RT) saline. Brains were then harvested and dissected to collect hippocampus, which was flash frozen in liquid nitrogen and then stored at −80°C. The standard housing group was five mice per cage with the exception for single housing during Morris water maze studies. All studies were performed on mice with a congenic C57BL/6J background. Cognitive and behavioral studies were conducted during the light cycle. The ages of the mice used are indicated in legends. Male mice were used in experiments. All cognitive, behavioral, and synaptic plasticity studies were conducted with the experimenter blinded to treatment. Studies were approved by the Institutional Animal Care and Use Committee of the University of California, San Francisco, and conducted in compliance with National Institutes of Health guidelines.

### Drug treatment.

Mouse KL1 (mKL1; catalog #DKOKO11705A, R&D Systems), human KL (hKL; catalog #SFN250712A, R&D Systems; lot #13669–819777, LakePharma), human KL1 (lot #14259–819582, LakePharma; lot #20190510–7093A, WuXi) were diluted in PBS, pH 7.5, and injected subcutaneously at a volume of 10 µl/g (adjusted to weight of mouse) at a dose of 10 µg/kg 4 h before behavior testing or sample collection for targeted metabolomic analysis. Proteins were coded to keep experimenters blind to the identity of proteins used in experiments. Recombinant proteins were used within 1 week of thawing from −80°C stock solutions and were stored at 4°C.

### Targeted metabolic profiling.

Hippocampal samples were analyzed at the Metabolite Phenotypes of Aging Core at the Nathan Shock Center at University of Washington for targeted and quantified metabolite measures. Samples were randomized and prepared as described previously (de Lucia et al., 2021). Internal quality control and sample quality controls were run alongside the study samples. Samples were analyzed by liquid chromatography tandem mass spectrometry (LC-MS/MS) at the Northwest Metabolomics Research Center, and the relative metabolite concentrations were reported as the peak areas. Data visualization and analysis were performed using custom R scripts and the “Glimma,” “limma,” “ggplot2,” “dlpyr,” “VISION,” “pheatmap,” “edgeR,” and “impute” Bioconductor packages were used. Briefly, the data were normalized to the protein concentration and then an EIGEN normalization was performed (Karpievitch et al., 2014) for normal distribution. Differentially expressed metabolites were identified using the standard limma pipeline, as described previously (Harrison et al., 2020). The *p* values were adjusted for multiple testing and metabolites with FDR. Principal component analysis (PCA) was used as the multivariate analysis to indicate the separation between the groups (ellipses at 95% confidence intervals), and further hierarchical clustering was performed using Ward's clustering algorithm to generate an expression heatmap. The biological significance of differentially expressed metabolites was explored using ingenuity pathway analysis (IPA, version 01-20-04) by identifying pathways that most closely associate with the differentially expressed metabolites. Data availabilty for metabolomics is available by R code on Github (https://github.com/Gupta-Shweta/Gupta-el-al-2022-Data-Repository).

### Electrophysiology.

Coronal brain slices of 300 μm thickness were obtained as described (Dubal et al., 2014, 2015; Leon et al., 2017), with some modifications, including measurements that were obtained from the CA1 region following stimulation of the Schaffer collateral path. Mice were anesthetized with 100% isoflurane, and the brain was collected and immediately placed in ice-cold artificial CSF (aCSF) containing the following (in mm): 124 NaCl, 2.8 KCl, 2 MgSO_4_, 1.25 NaH_2_PO_4_, 10 glucose, 26 NaHCO_3_, 2.5 CaCl_2_, and 1.3 ascorbic acid; and sliced on a vibratome (model VT1200, Leica). Slices were incubated at 32°C for 30 min and then incubated at RT for 1 h before testing. Slices were transferred to an interface chamber with circulating oxygenated (95% O_2_ and 5% CO_2_) aCSF at 30°C and were left to recover for 10–15 min before any stimulation. For field potential recordings, acute hippocampal slices were placed on a multielectrode array (model Med64-Quad II, Alpha MED Scientific). Field EPSPs (fEPSPs) were elicited and recorded via planar electrodes of the Quad II 2 × 8 probe (model AL-MED-PG501A, Alpha MED Scientific) by aligning the electrodes and the stratum radiatum region of hippocampal slices. An input–output curve was performed at the beginning of each recording to determine the appropriate stimulation intensity. Test stimuli at 35% of maximal intensity were delivered at 0.05 Hz, and a stable baseline fEPSP at 15–20 min was established before long-term potentiation (LTP) induction. LTP was induced using a theta-burst protocol comprised of two trains delivered every 20 s, with each train containing 10 bursts at 5 Hz, and each burst containing four pulses at 100 Hz. Recordings and analysis were performed using Med64 Mobius Software (Alpha MED Scientific). Following stable LTP induction, the last 10 min of a usual 60 min recording was averaged to compare the experimental groups.

### Small Y maze.

Mice were tested in the small Y maze, also known as spontaneous alternation Y maze with spatial cues, as previously described (Dubal et al., 2014, 2015; Leon et al., 2017; Davis et al., 2020). Briefly, mice were placed in the testing room 1 h prior for acclimation. For each test, a mouse was placed inside one of the three identical arms of the Y maze and was allowed to explore the apparatus for 6 min. The maze was covered with a clear plastic board, and recording began. The mouse was returned to its home cage, and the apparatus was cleaned with 70% ethanol between trials. The spontaneous alterations were scored by hand while watching the video, and the percentages of alternations were calculated.

### Morris water maze.

Mice were tested in the Morris water maze as described previously (Dubal et al., 2014, 2015; Leon et al., 2017; Davis et al., 2020). Briefly, the water maze pool (diameter, 122 cm) contained white, opaque water (21 ± 1°C) with a small, square, 10 cm^2^ platform submerged and hidden 2 cm below the surface. Distinct visual cues were present on three walls in the testing room. Before hidden platform training, mice underwent two pretraining trials by swimming through a channel to mount a hidden rescue platform. During hidden platform training, the platform location remained constant, and the drop location varied between trials. Mice received two training sessions, consisting of two trials each, daily for 4 d. The maximum time allowed per trial was 60 s. For the probe trial, the platform was removed, and the mice were allowed 60 s to search. Following probe testing, mice were tested for their ability to find the platform when marked with a visible cue (15 cm pole placed on the visible platform). Scoring was performed using EthoVision XT automated software (Noldus) according to the standard instructions.

### Two-trial Y maze.

Mice were tested in the two-trial Y maze, which assesses spatial and working memory, as previously described (Leon et al., 2017). Briefly, distinct visual cues were attached to the ends of the novel and familiar arms, while the start arm was left blank. During training, one of the two visual cue arms was blocked (novel arm). Mice were then placed in the start arm and allowed to explore the two open arms (start and familiar) freely for 5 min. The novel and familiar arm designation was alternated between mice tested to control for any innate preference mice might have for one arm. After training, mice were returned to their home cage. Sixteen hours later, mice were tested with all three arms unblocked. Mice were allowed to explore freely for 5 min, and the duration of time, as well as the distance traveled in the novel and familiar arms were measured automatically using the ANY-Maze system (San Diego Instruments).

### Statistics.

Statistical analyses were conducted with GraphPad Prism (version 7.0) for *t* tests and ANOVAs. Two-tailed *t* tests were used to determine differences between two means, and two-way ANOVA was used to determine differences between multiple means. R (version 4.0.2) was used for Pearson's correlation analysis and *post hoc* tests. A mixed-model ANOVA was used for the Morris water maze data analysis and included effects of repeated measures. *Post hoc* tests are indicated in the legends. In mouse studies of behavior and synaptic plasticity, exclusion criteria (>2 SDs above or below the mean) were defined a priori to ensure unbiased exclusion of outliers. Error bars represent +SEM. Null hypotheses were rejected at or below a *p* value of 0.05. For metabolomic data analysis, linear mixed-effect models were fit using the standard lme4 package in R.

## Results

### Cognitive stimulation remodels the metabolome of the hippocampus

**Figure 1.**
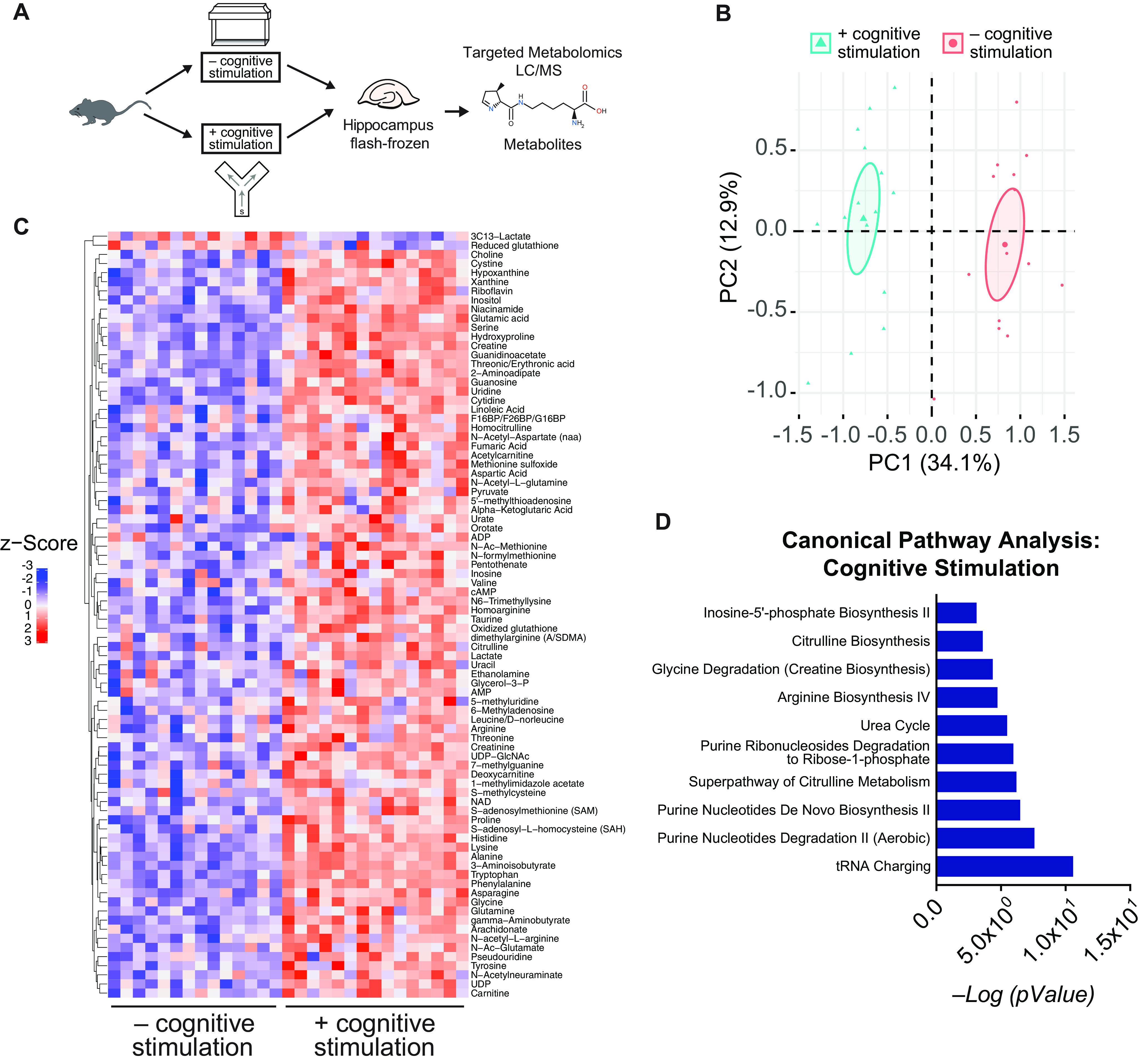
Brain engagement, through a cognitive task, remodels the hippocampal metabolome. ***A***, Experimental paradigm for performing targeted metabolomic analysis following cognitive challenge. Male mice (age, 4 months; *n* = 14–16/experimental group) underwent a cognitive challenge (exploration of a small Y maze with spatial cues) or exploration of their home cage. The hippocampus was then immediately collected and flash frozen, and then underwent targeted metabolomic profiling. ***B***, PCA plot of normalized counts of metabolites extracted from hippocampus show a clear separation between the hippocampi of mice with or without a cognitive challenge. ***C***, Heatmap clustering analysis representing all the metabolites that were significantly different between mice with and without a cognitive challenge. Data were EIGEN normalized, log transformed, and autoscaled. ***D***, Pathway analysis using IPA of the 84 metabolites that significantly differed included purine metabolism, amino acid metabolism, and citrulline metabolism among most significantly associated pathways PC, principle component.

We first assessed whether task engagement, or cognitive stimulation, alters the metabolome of the hippocampus, a central region for learning and memory. Mice explored either a small Y-maze with spatial cues, which measures working and spatial memory, or their home cage for 5 min followed by immediate hippocampal collection. We performed targeted metabolomics of the hippocampi using LC-MS/MS against a panel representing 42 metabolic pathways that quantitatively detected 154 metabolites (Fig. 1*A*). PCA showed that levels of metabolites clearly distinguished the two experimental groups from each other (Fig. 1*B*). Cognitive stimulation significantly upregulated 82 metabolites and downregulated 2 metabolites in the hippocampus, as shown in the heatmap (Fig. 1*C*). A canonical pathway analysis using IPA indicated that cognitive stimulation engaged purine and amino acid metabolism as overarching pathways in the hippocampus (Fig. 1*D*). Among the metabolites in these pathways, many, including arginine, citrulline, and dimethylarginine, decrease in the aging brain (Gupta et al., 2012; Zheng et al., 2016) and in models of neurodegenerative disease (Fleszar et al., 2019), suggesting that the inverse, or metabolite increase, may be beneficial for the brain. Thus, cognitive stimulation induced distinct pathways and largely increased metabolite levels in the hippocampal metabolome.

### Longevity factor KL, mediated by its subdomain KL1, mimics the metabolic signature of cognitive stimulation

**Figure 2.**
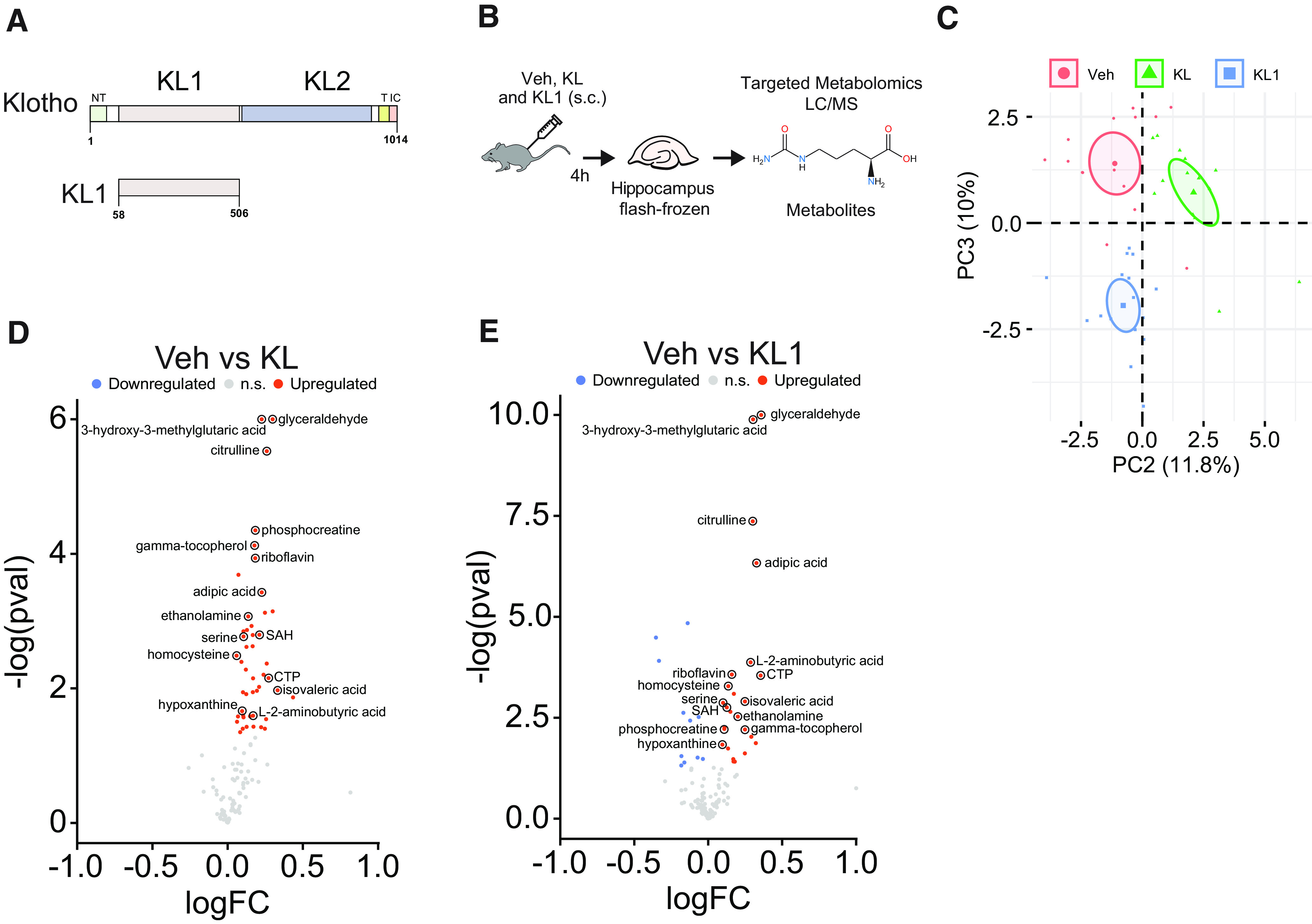
KL and its KL1 subdomain alter the hippocampal metabolome. ***A***, Diagram of the full-length klotho protein (top, amino acids 1–1014) including its subdomains and (bottom, amino acids 58–506) the KL1 protein. ***B***, Experimental paradigm for performing targeted metabolomic analysis of mice that were treated with vehicle (Veh), human KL (10 μg/kg, s.c.), or human KL1 (10 μg/kg, s.c.). Male mice (age, 4 months; *n* = 12–13/experimental group) were treated. Four hours later, hippocampi were collected, flash frozen, and then underwent targeted metabolomic profiling. ***C***, PCA plot of normalized counts of metabolites extracted from hippocampus shows clear separation among the three treatment groups on components 2 and 3. ***D***, Volcano plot showing the significantly differentially expressed metabolites in the KL- compared with Veh- treated group. ***E***, Volcano plot showing the significantly differentially expressed metabolites in the KL1- compared with the Veh-treated group. In ***D*** and ***E***, the 15 labeled metabolites are all shared between KL- and KL1-treated groups. PC, principal component.

Since KL actions on cognition are rapid, as are metabolic changes, we tested whether KL treatment, through its KL1 subdomain (Fig. 2*A*), alters the hippocampal metabolome, and whether this alteration overlaps with that of cognitive stimulation. To this end, we performed targeted hippocampal metabolomics on mice 4 h following injection with vehicle, recombinant human KL protein (10 μg/kg, s.c.), or recombinant human KL1 (10 μg/kg, s.c.; Fig. 2*B*). We chose this time point since KL rapidly enhances cognition by 4 h following treatment (Leon et al., 2017). We used human forms of the proteins for metabolomics since they might be more translationally relevant tools in the context of the human condition. PCA showed that metabolite levels distinguished the three treatment groups from each other (Fig. 2*C*). KL-treated mice compared with vehicle-treated mice showed 41 differentially abundant metabolites, all upregulated (Fig. 2*D*). KL1-treated mice compared with vehicle-treated mice showed 27 differentially abundant metabolites (16 upregulated and 11 downregulated) (Fig. 2*E*) KL- and KL1-treated mice shared the upregulation of 15 hippocampal metabolites, as labeled on the volcano plots (Fig. 2*D,E*). Thus, KL and KL1 treatments acutely altered the hippocampal metabolome, within the short time frame of 4 h that KL enhances cognition (Leon et al., 2017).

**Figure 3.**
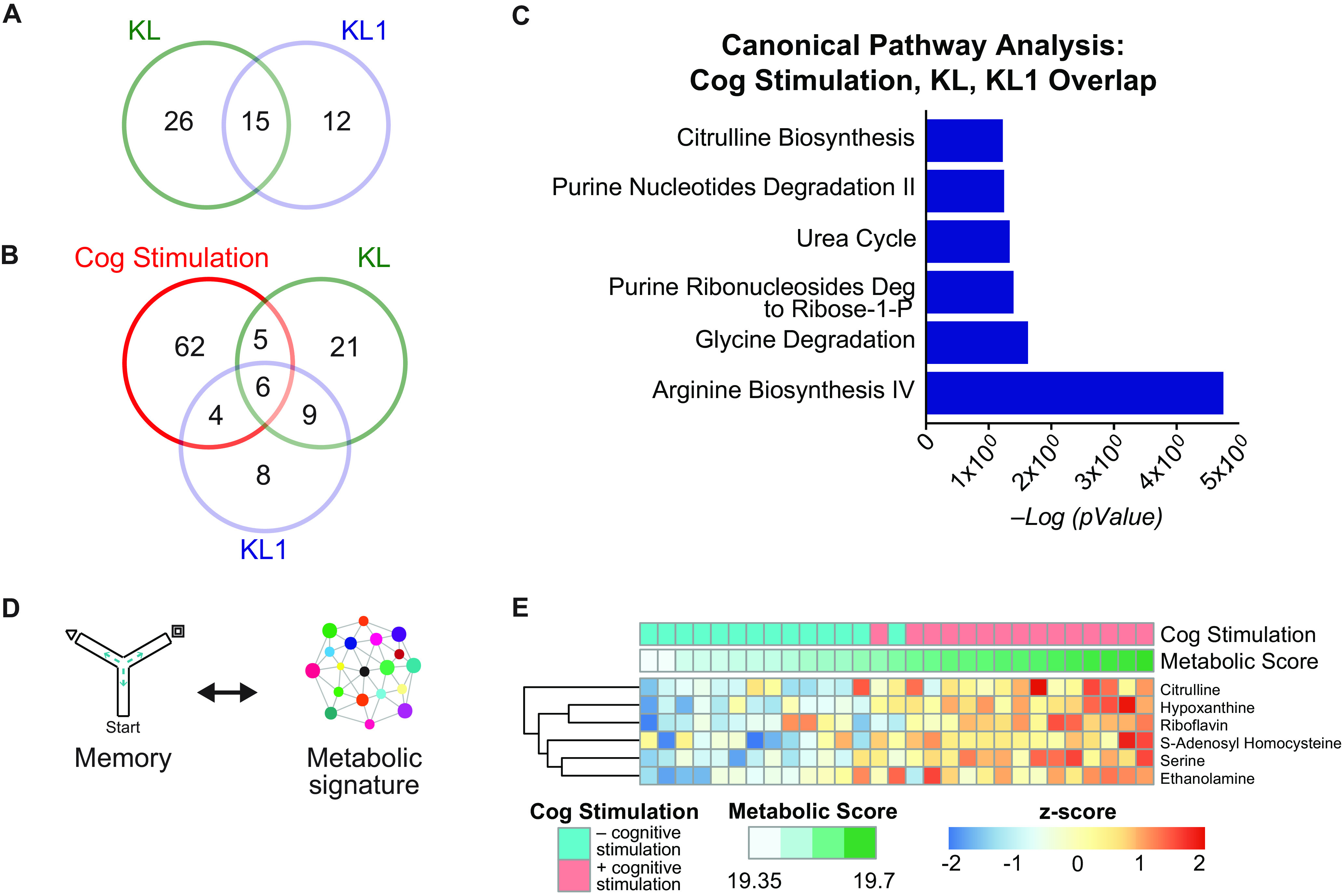
KL and KL1 partially mimic the hippocampal metabolome of cognitive (Cog) stimulation and correlate with cognitive performance. ***A***, Venn diagram showing the overlap between differentially expressed metabolites between KL- and KL1-treated mice. Over 50% of klotho-mediated metabolic changes were recapitulated with KL1 treatment. ***B***, Venn diagram showing the overlap among differentially expressed metabolites in the cognitively stimulated, KL-treated, and KL1-treated mice. Twenty-seven percent of KL-induced metabolites and 37% of KL1-induced metabolites overlapped with cognitive stimulation. ***C***, IPA canonical pathway analysis of the overlapping metabolites among cognitive stimulation, KL, and KL1 shows that a subset (shown) are identical metabolic pathways compared with cognitive stimulation. ***D***, Diagram of approach of mapping cognitive performance to hippocampal metabolomics to generate a metabolic signature of cognition. ***E***, Heatmap of the signature of six common metabolites shared among cognitive stimulation, KL treatment, and KL1 treatment. The signature was used to generate a metabolic score, and cognitively stimulated mice showed an increased metabolic score. Deg, Degradation.

We next assessed whether KL- and KL1-mediated metabolic changes overlap with the hippocampal metabolome following cognitive stimulation. Around 55% of KL1-mediated metabolic alterations overlapped with those of full-length KL (Fig. 3*A*), indicating that the KL1 subdomain captures a majority of the metabolic alterations of KL. Furthermore, KL and KL1 treatment each induced differentially abundant metabolites that overlapped 27% and 37%, respectively, with the metabolome of cognitive stimulation (Fig. 3*B*). Thus, in the absence of a cognitive task, KL and KL1 treatment caused metabolic alterations that partially mimicked a cognitively stimulated brain.

Pathway analysis of overlapping metabolites among cognitive stimulation, KL, and KL1, revealed shared pathways of amino acid and purine metabolism (Fig. 3*C*), which are also prominent in cognitive stimulation itself (Fig. 1*D*). We then mapped the six overlapping, differentially abundant metabolites to cognitive performance of individual mice (Fig. 3*D*). We generated a metabolic score for each mouse derived from the abundance levels of the shared metabolites, and a memory score derived from mice that explored the small Y maze with spatial cues. As expected, cognitive stimulation increased the metabolic score (Fig. 3*E*). The metabolic score correlated with the memory score (*R*^2^ = 0.51, *p* = 0.05), indicating enhanced cognitive functions with higher metabolic scores. Thus, KL- and KL1-mediated mimicry of cognitive stimulation suggests that their metabolic remodeling of the hippocampus could prime the brain for improved cognition.

#### Klotho subdomain KL1, in mouse and human forms, is sufficient for synaptic and cognitive enhancement in young mice

**Figure 4.**
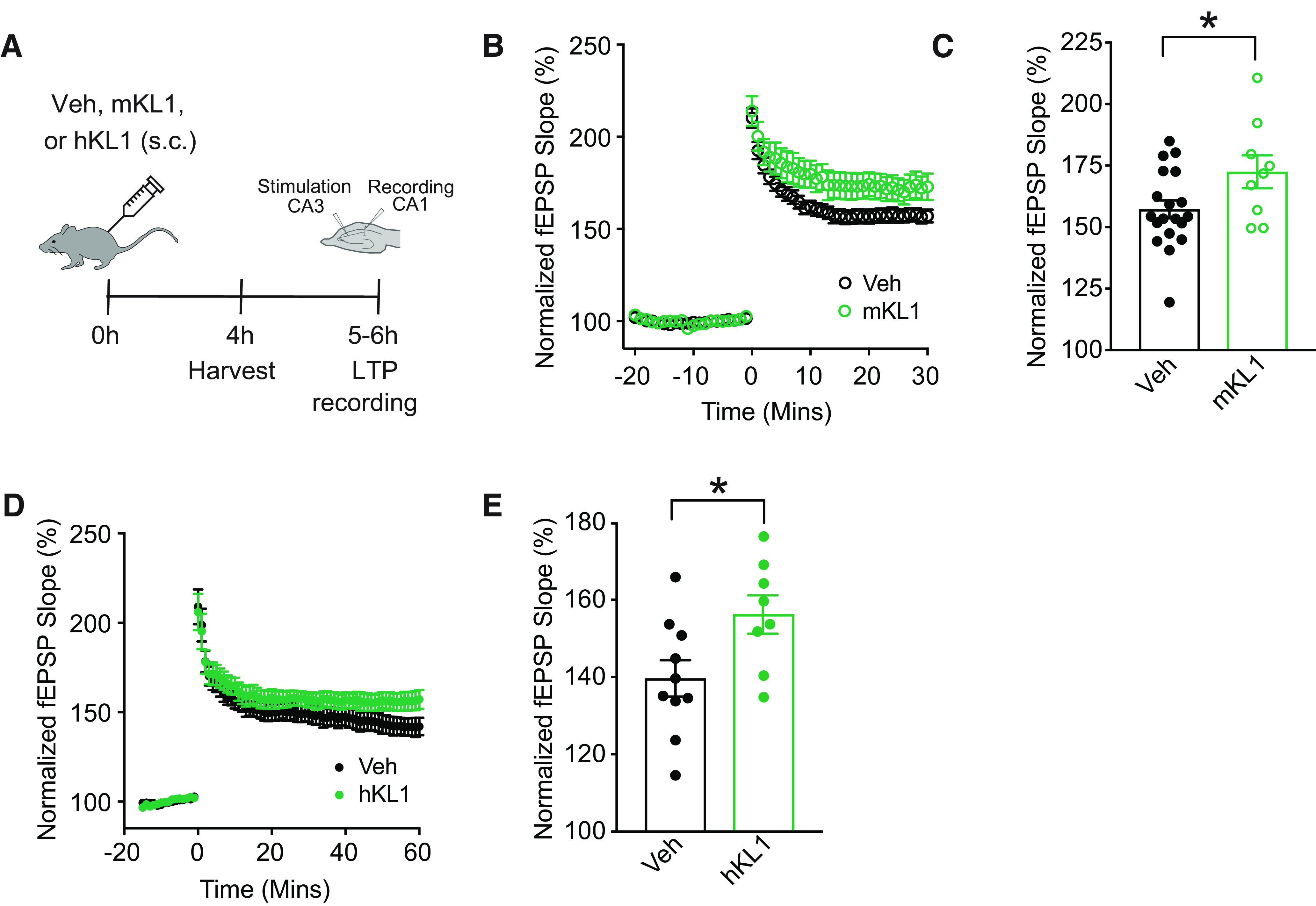
KL1 increases synaptic plasticity. ***A***, Experimental paradigm of hippocampal LTP recordings from male mice (age, 3 months) treated with vehicle (Veh) or KL1 (mouse or human form: 10 μg/kg, s.c.). ***B***, fEPSP recordings from acute hippocampal slices of mice treated with vehicle or mouse KL1 (*n* = 19 slices from five mice for vehicle; 9 slices from three mice for mKL1). ***C***, Average fEPSP slope of the last 10 min (Mins) of each recording for vehicle- and mKL1-treated mice. *p = 0.0377, df = 26 (two-tailed *t* test). Values represented are data ± SEM. ***D***, fEPSP recording from acute hippocampal slides of mice treated with vehicle or hKL1 (*n* = 10 slices from three mice for vehicle; 8 from three mice for hKL1). ***E***, Average fEPSP slope of the last 10 min of each recording for vehicle- and hKL1-treated mice. *p = 0.0285, df = 16. Values represented are data ± SEM Veh, vehicle. mKL1, mouse KL1. hKL1, human KL1.

Cognitive stimulation (Aidil-Carvalho et al., 2017) and KL (Dubal et al., 2014, 2015; Leon et al., 2017; Zeng et al., 2019) both improve brain functions; however, whether KL1—a KL subdomain and closer mimic of cognitive stimulation to the metabolome—is sufficient to influence cognition is unknown. We first tested whether KL1 can enhance synaptic plasticity, in the form of LTP, a key substrate of learning and memory (Morris et al., 1986; Nakazawa et al., 2004). We assessed LTP in acute hippocampal slices in the CA1 Schaffer collateral pathway synapse. Acute treatment with KL1, in both the mouse (mKL1) and human (hKL1) species forms (94% conserved) enhanced synaptic plasticity in the normal adult brain (Fig. 4*A–E*).

**Figure 5.**
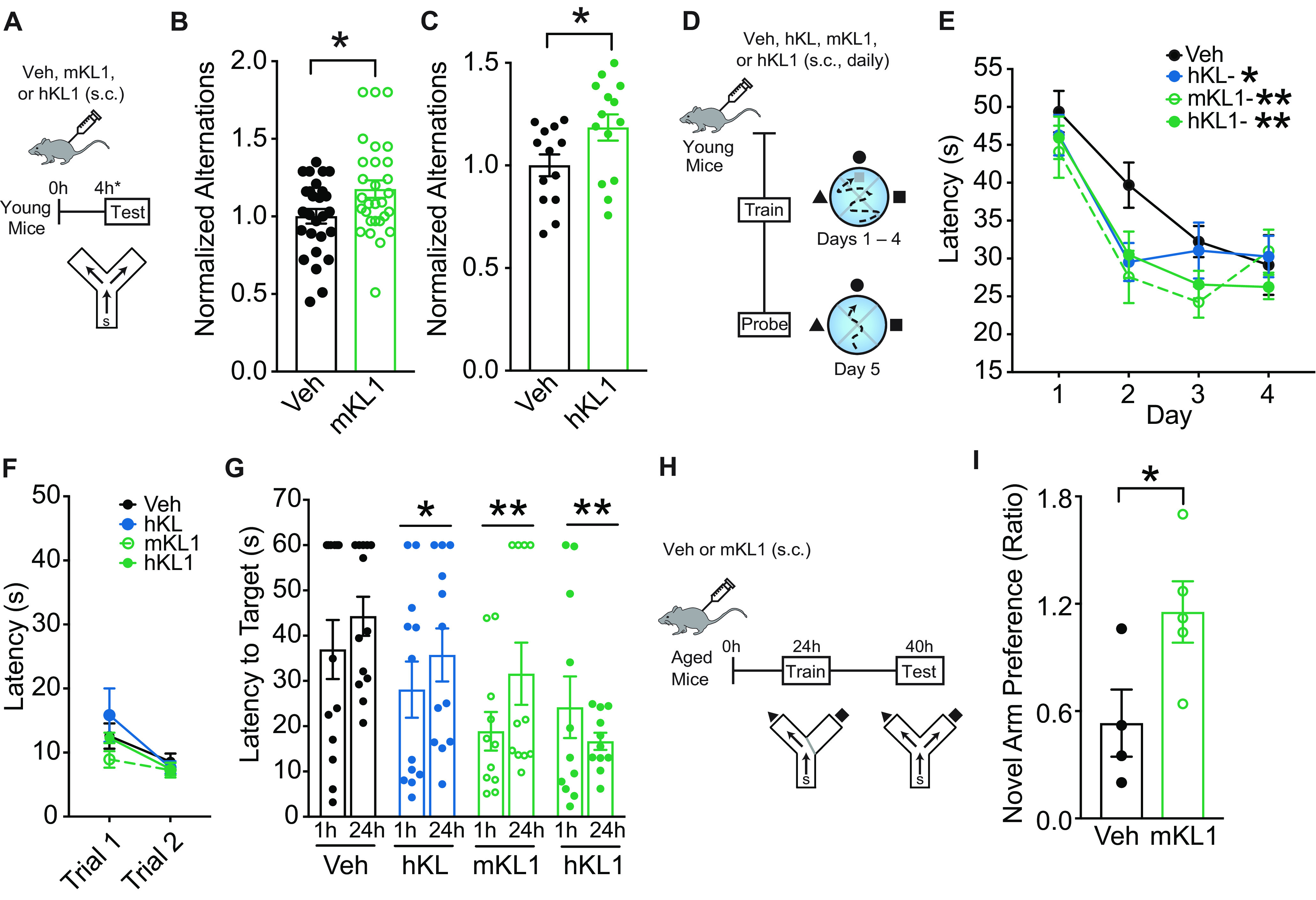
KL1 enhances cognition in young mice and counters cognitive deficits in old mice. ***A***, Experimental paradigm for testing mice (age, 4 months) in the small Y maze following treatment with vehicle (Veh) or KL1 (mouse or human form: 10 μg/kg, s.c.). ***B***, Alternations of mice treated with vehicle or mKL1 (*n* = 27–29/experimental group). Data expressed relative to control group. *p = 0.0212, df = 54 (two-tailed *t* test). ***C***, Alternations of mice treated with vehicle or hKL1 (*n* = 13–14 mice/experimental group). Data are expressed relative to control group. *p = 0.0372, df = 25 (two-tailed *t* test). ***D***, Experimental paradigm for testing spatial learning and memory of mice in the Morris water maze. Mice were injected daily with vehicle, hKL (10 μg/kg, s.c.), mKL1 (10 μg/kg, s.c.), and hKL1 (10 μg/kg, s.c.). ***E***, Spatial learning curves (platform hidden) in the water maze in mice treated with vehicle, hKL, mKL1, and hKL1 (*n* = 11–13 mice/experimental group). Data represent the daily average of the latency (in seconds) to find the location of the hidden platform. All KL and KL1 groups enhanced learning compared with vehicle group (two-way repeated-measures ANOVA): Treatment: *p* = 0.0071, *F*_(3,44)_ = 4.577; Veh versus hKL (*q = 0.0223); Veh versus mKL1 (**q = 0.0020); Veh versus hKL1 (**q = 0.0034; *post hoc* corrected *q* values, Benjamini, Krieger, and Yekutieli test). ***F***, Trials with platform visible, showing no differences in the ability to find a visible target among experimental groups. ***G***, Probe trials conducted 1 and 24 h after hidden training and removal of the escape platform. KL and KL1 (mouse or human) decreased latency to find the target platform, indicating memory enhancement (*n* = 11–13 mice/experimental group). Two-way repeated-measures ANOVA: Treatment: *p* = 0.0072, *F*_(3,43)_ = 4.582; Veh versus hKL (*q = 0.0464); Veh versus mKL1 (**q = 0.0059); Veh versus hKL1 (**q = 0.0012; *post hoc* corrected *q* values, Benjamini, Krieger, and Yekutieli test). ***H***, Experimental paradigm for testing working and spatial memory of old mice (age, 22 months) in the two-trial Y maze. Old mice were injected with vehicle or mKL1 (10 μg/kg, s.c.) and then underwent testing and training. ***I***, Spatial and working memory of young and aging mice treated with Veh or mKL1 was assessed by the two-trial Y maze (*n* = 4–5/experimental group). mKL1 increased the ratio of the percentage of time spent in the novel versus familiar arms during testing at 3 min, indicating improved memory. *p = 0.0450, df = 7 (two-tailed *t* test). Values represented are data ± SEM. Veh, Vehicle. mKL1, mouse KL1. hKL1, human KL1. hKL, human KL.

To assess whether the KL1-induced metabolic alterations could prime the brain for better function, we next directly tested whether KL1 enhances cognition. We tested mice in the small Y maze, which measures working and spatial memory. Young adult (age, 3–4 months) mice were treated with vehicle or KL1 (mouse or human; 10 μg/kg, s.c.) and 4 h later explored the small Y maze with spatial cues (Fig. 5*A*). Both mouse (Fig. 5*B*) and human (Fig. 5*C*) KL1 immediately enhanced cognition in this task. We then tested a separate cohort of mice in the Morris water maze, which measures spatial learning and memory (Fig. 5*D*). Young mice were treated with vehicle, human KL (10 μg/kg, s.c.), mouse KL1 (10 μg/kg, s.c.), or human KL1 (10 μg/kg, s.c.) daily. As expected (Leon et al., 2017), KL enhanced learning, which was measured by decreased latency to find the hidden platform (Fig. 5*E*). In addition, both human and mouse KL1 also enhanced learning (Fig. 5*E*). Mice showed no differences in swim speeds or the ability to find target platform in any of the experimental groups (Fig. 5*F*), indicating the specificity of the findings to learning and memory. In a probe trial to test memory of the hidden platform location, 1 and 24 h after training, all KL and KL1 treatment groups showed enhanced memory (Fig. 5*G*). Thus, mouse and human forms of KL1 enhanced learning and memory, and KL1, which circulates endogenously, recapitulated KL-mediated cognitive enhancement in the normal brain. Notably, both mouse and human forms of KL1 similarly enhanced cognition, suggesting that either can be used in mouse experiments.

### KL1 counters cognitive decline in aging

Since aging is a major cause of cognitive deficits, an unmet biomedical challenge, we tested whether KL1 treatment could counter cognitive deficits in aging. Aging preferentially targets spatial and working memory; therefore, we tested mice in a two-trial Y maze that measures these domains and is sensitive to effects of aging (Bizon et al., 2012). Old mice (22 months) were treated once with either vehicle or mouse KL1 (10 μg/kg, s.c.); 24 h later, they underwent training, and 40 h later, testing (Fig. 5*H*). KL1 increased exploration of the novel compared with familiar arm (Fig. 5*I*), indicating that it improved cognition in the aging brain. Collectively, these findings show that the KL1 protein, a subdomain of KL that mimics the effects of cognitive stimulation on the metabolome, enhanced cognition in young and aged mice, and was sufficient to recapitulate KL-mediated cognitive enhancement.

## Discussion

Our metabolomic, informatic, electrophysiologic, pharmacologic, and behavioral studies in mice collectively reveal that cognitive stimulation and longevity factor KL, mediated by its KL1 subdomain, shared convergent and overlapping roles in acutely remodeling the brain metabolome; within the same rapid time frame of these robust hippocampal alterations, KL1 increased cognitive functions. Cognitive stimulation increased amino acid and purine metabolism in the mouse hippocampus. Acute treatment with longevity factor KL, an intervention that enhances cognition, mimicked the hippocampal metabolome of cognitive stimulation, in the absence of a behavioral task. Importantly, the KL1 subdomain of KL, which was a closer mimetic of the metabolome of cognitive stimulation than KL alone, was sufficient to increase synaptic plasticity, enhance normal cognition, and counter cognitive deficits in aging. Together, these data support the hypothesis that KL treatment mimics the effects of cognitive stimulation and enhances the brain through its subdomain KL1.

Engagement of the brain through cognitive stimulation is a behavioral intervention that improves cognition (Wilson et al., 2002; Gates et al., 2011) and is known to require energy; but how it metabolically changes the brain remains largely unknown. Our finding that it induces multiple metabolites potentially reflects the repletion of energy used during cognition, stimulation of metabolic pathways that improve cognition, or both. Our data, in part, support the latter possibility since KL1 treatment also induced similar metabolites in the absence of a cognitive task, and, in the same 4 h time frame as its metabolic alterations, enhanced synaptic plasticity and cognition. It remains to be determined how long KL- and KL1-induced metabolic alterations last and whether they may initiate other signaling pathways. Understanding the metabolome of cognitive stimulation opens the path to mimic its effects and enhance brain functions.

Brain engagement through cognitive stimulation robustly increased purine and amino acid metabolism in the hippocampus. KL, a longevity factor, and its KL1 subdomain recapitulated these metabolic alterations. Curiously, other interventions that promote longevity, such as reduced insulin/IGF-1 signaling and caloric restriction (which, similar to intermittent fasting, also elevates KL; Shafie et al., 2020; Dias et al., 2021), likewise increase amino acid and purine metabolism (Gao et al., 2018) in *Caenorhabditis elegans*, producing a metabolic signature in the body similar to that of cognitive stimulation and KL treatment in the brain. The convergence of interventions that improve life span and brain health in amino acid and purine metabolism suggests common metabolic pathways to promoting better brain health.

Cognitive stimulation probably influenced astrocyte metabolism since astrocytes are the main source and site of purine (Ciccarelli et al., 2001) and citrulline (Lerchundi et al., 2015; Bonvento and Bolaños, 2021) biosynthesis, both of which have been observed to increase with cognitive stimulation. Further, astrocytes harbor pools of energy produced from metabolic pathways that are transferred to neurons (Schousboe et al., 2013). It should be noted that, since targeted metabolomics was performed on the whole hippocampus, cell type-specific metabolomic responses, such as astrocytic changes observed in injury to the blood–brain barrier (BBB; Huang et al., 2020), were not able to be discerned.

Cognitive stimulation and treatment with KL or KL1 shared a hippocampal metabolic signature that associated closely with improved cognitive performance in individual mice. This means that cognition improved with increased levels of the combination of metabolites captured by the metabolic score. While one cannot assume causation between the variables, a growing body of evidence supports causal effects of several of the metabolites. Among them, it is interesting to note that serine, an amino acid and coagonist of NMDA receptors, improves synaptic plasticity in the normal brain and in models of AD (Le Douce et al., 2020). Similarly, citrulline, an amino acid and intermediate of the urea cycle, counters age-induced deficits of synaptic plasticity (Ginguay et al., 2019). Further, riboflavin, also known as vitamin B2, which is necessary for metabolic energy production, counters cognitive deficits in AD model mice (Zhao et al., 2018). Collectively, this suggests beneficial outcomes from selective elevation of hippocampal metabolites within the signature.

The KL1 subdomain is half the molecular size of KL and was sufficient for cognitive enhancement. Because KL is a pleiotropic protein that acts throughout the body and engages multiple signaling pathways, narrowing the domain for neural functions may facilitate a targeted and simpler KL mimetic. Indeed, KL1-mediated signaling pathways are narrower than KL. For example, while KL stimulates FGF-23 signaling (Chen et al., 2018), KL1 does not (Château et al., 2010), as expected by its location far from the FGF receptor binding site within the atomic structure of KL (Chen et al., 2018). However, like KL, KL1 binds α2-3-sialyllactose, thus predicting sialidase activity (Wright et al., 2017), an enzymatic function of KL that contributes to cellular signaling. Whether KL1 acts via its putative sialidase activity to stimulate hippocampal amino acid and purine metabolism, and how this metabolism specifically links with enhanced cognition, remain to be determined.

Acute, systemic elevation of KL, as in the current experiments, increases synaptic plasticity, cognition, and neural resilience without crossing into the brain (Dubal et al., 2014; Hu et al., 2016). However, it remains unknown whether KL1 could cross the BBB. We speculate that both KL and KL1 engage peripheral messengers that transduce yet unidentified signals into the brain. Alternatively, if transported across the BBB, KL1 itself might be a messenger of KL.

We assessed both the human and mouse forms of KL1, along with the human form of KL, and found similar efficacies in synaptic and cognitive enhancement among these proteins. This indicates that the 94% conservation between the mouse and human KL1 sequences preserves biological functions related to cognition, and that either species of KL1 protein can be useful in future studies. An advantage of using human forms of the proteins is they may serve as translationally relevant tools to study in the context of the human condition.

Further investigation into the metabolome of cognitive stimulation, along with understanding how KL1 mimics this metabolome and ultimately improves brain function, opens pathways to pharmacologic interventions to enhance normal cognition and counter cognitive deficits from aging and disease.
